# Almond supplementation reduces serum uric acid in coronary artery disease patients: a randomized controlled trial

**DOI:** 10.1186/s12937-016-0195-4

**Published:** 2016-08-19

**Authors:** Humaira Jamshed, Anwar-ul-Hassan Gilani, Fateh Ali Tipoo Sultan, Faridah Amin, Jamshed Arslan, Sumaira Ghani, Madiha Masroor

**Affiliations:** 1 Department of Biological and Biomedical Sciences, Aga Khan University, Karachi, Pakistan; 2 Pakistan Council for Science and Technology, Government of Pakistan, Shahara-i-Jamhuriat, G-5/2, Islamabad, Pakistan; 3 Department of Medicine, Aga Khan University, Karachi, Pakistan

**Keywords:** Hyperuricemia, Coronary artery disease, Nuts, Soaked almonds, Low dose

## Abstract

**Objective:**

Elevated serum uric acid (UA), a biomarker of renal insufficiency, is also an independent prognostic marker for morbidity in coronary artery disease (CAD) and poses serious health risks. This study reports the effect of almond consumption on UA in CAD patients.

**Study design:**

A randomized controlled clinical trial was conducted with three groups: no-intervention (NI), Pakistani almonds (PA) or American almonds (AA). Patients were recruited from the Cardiology Clinics, Aga Khan University Hospital. Two follow-ups were scheduled at week-6 and week-12. 150 patients were randomly divided in three groups (50 per group). NI was not given almonds, whereas the PA and AA were given Pakistani and American almond varieties (10 g/day), respectively; with instruction to soak overnight and eat before breakfast.

**Results:**

Almonds supplementation significantly reduced (*p* < 0.05) serum UA among groups, and over time. At week-6, UA concentrations were -13 to -16 % less in PA and AA; at week-12 the concentrations were -14 to -18 % less, compared to NI. Systolic and diastolic blood pressure and body weights of the participants remained fairly constant among all the groups.

**Conclusion:**

Almonds (10 g/day), eaten before breakfast, reduces serum UA in CAD patients. Prevention of hyperuricemia can confer protection from kidney and vascular damage and if extrapolated for general population, dietary almonds can offer grander health benefit. Trial is registered at Australian New Zealand Clinical trial registry as ACTRN12614000036617.

## Introduction

There has been considerable increase in global prevalence of hyperuricemia, in the past few years, backed by western dietary patterns. Where higher serum uric acid (UA) frequently indicates renal insufficiency [1], it may also be associated with coronary artery disease (CAD) [2], and deliberated as a prognostic marker for morbidity and mortality, independent of other risk factors [3]. Even in patients with no history of heart disease or stroke, elevated UA was associated with higher risk of myocardial infarction or stroke [4]. An increase in serum UA by 1 mg/dL contributes to 12 % increase in risk of CAD death [5]. This association is believed to be stronger in women than men [5]. But there are also some indications where, in middle-aged men, hyperuricemia served as a risk factor for cardiovascular and all-cause mortality [6].

UA is shown to possess anti-oxidant potential [7], which undermines its causative role in chronic diseases. Yet, its establishment as a comorbid risk marker is backed by recent meta-analysis [8] and systemic review [5] showing significant correlation between hyperuricemia and CAD. Further signifying its role for cardiovascular health, are the studies where anti-hypertensive and/or lipid-neutralizing therapies limited UA production, thereby reducing CAD mortality [9, 10].

Almonds are among the nuts approved by Food and Drug Administration – United States to have the potential of reducing CVD risk [11]. The lipid-neutralizing properties, among others, have been extensively elaborated [12]. Dietary supplementation of almonds is shown to prevent hyperuricemia in a cardiovascular disease rat model [13]. This study inspects the UA-reducing potential of almond supplementation in CAD patients.

## Methods

### Study population

Hundred and fifty CAD patients were recruited from Cardiology Clinic at The Aga Khan University Hospital, Karachi. The eligibility criteria and trial logistics have previously been defined in detail, following the CONSORT guidelines [14]. Briefly, patients consuming nuts on regular basis (>15 g/d; three days/week) or those having nut allergies were excluded. Informed consent was obtained. Using block randomization, patients were assigned any of the following three groups: No Intervention (NI); Intervention with Pakistani almonds (PA) or American almonds (AA).

### Study design

Approval was obtained from Ethical Review Committee of The Aga Khan University, Karachi, Pakistan (Application ID: 2230-Med-ERC-12), and the trial was registered at Australian New Zealand Clinical Trial Registry (ID: ACTRN12614000036617).

Baseline blood was drawn and vitals (body weight, blood pressure etc.) were recorded. Participants of NI were instructed not to consume any nuts, specifically almonds, during their enrollment in trial; whereas participants of PA and AA were given almonds (10 g/day), with the instruction to consume in a traditional way (i.e. soak overnight, peel and eat before breakfast in the morning). Diaries, to record almond consumption, were provided. Compliance was monitored through regular phone calls (twice per week). Follow-up visits were scheduled at week-6 and week-12 (±3 days), at which blood samples were collected and vitals were recorded. Participants of NI received almonds after the completion of trial.

Pakistani almonds, namely Talwar, grown in Balochistan – Pakistan; and imported American almonds, locally available at Utility stores in Karachi, were used. Serum concentration of uric acid (UA) was measured on Roche Cobass c-111 automated analyzer using commercially available kit (Uric acid kit no. 4657608190).

Data were analyzed on SPSS version 17.0 and Graphpad Prism, and results are presented as means ± SEM. Two-way repeated measures ANOVA was used to compare means of groups followed by Bonferroni post-tests. For categorical data, chi-square test was used to compare differences between groups. *P*-value < 0.05 was considered statistically significant (95 % Confidence interval).

## Results

The flow of participants through the trial, as per CONSORT format, has been reported previously [14]. Attrition rate was around 15 %. The major reasons included: failure to contact (*n* = 20); leaving city in summer vacations (*n* = 8); and a few cases of angioplasty (*n* = 3). The baseline characteristics of participants in each group, is provided in Table 1. Systolic and diastolic BP and body weight of the participants remained fairly constant (*p* > 0.05) throughout the twelve weeks of the study, as seen in Table 2.

**Table 1:** Baseline characteristics of CAD patients randomized into no-intervention, Pakistani almond or American almond groups

	No Intervention^1^		Pakistani Almonds^1^		American Almonds^1^	
	Mean	SEM	Mean	SEM	Mean	SEM
Age, years	61	0.2	57	1.6	61	1.5
Gender						
Male, *n*	39		36		38	
Female, *n*	11		14		12	
Body weight, kg	73.4	0.2	79	1.9	75	1.6
Blood Pressure, mmHg						
Systolic	127	0.4	126	2.4	128	2.6
Diastolic	70	0.2	67	1.2	68	1.3
Serum Uric Acid, mg/dL						
Male	7.2	0.49	6.9	0.23	6.8	0.22
Female	5.9	0.34	5.7	0.14	5.6	0.21

^1^ *n* = 50 per group

**Table 2:** Blood pressure and serum uric acid concentrations of CAD patients randomized into no-intervention (NI), Pakistani almonds (PA) and American Almonds (AA) groups, at week-6 and week 12

		No Intervention				Pakistani Almonds				American Almonds			
		Male		Female		Male		Female		Male		Female	
		*n* = 25		*n* = 9		*n* = 27		*n* = 11		*n* = 33		*n* = 8	
		Mean	SEM	Mean	SEM	Mean	SEM	Mean	SEM	Mean	SEM	Mean	SEM
Blood Pressure													
Systolic, mmHg	Week 6	128	0.5	129	0.6	126	1.7	127	0.9	127	1.7	126	1.4
	Week 12	126	1.2	127	0.5	124	3.1	126	1.2	125	3.4	125	0.5
Diastolic, mmHg	Week 6	71	0.3	69	0.4	67	0.5	68	0.5	67	0.2	66	0.8
	Week 12	70	1.8	69	1.0	66	1.2	67	1.1	67	1.0	67	0.2
Uric Acid, mg/dL	Week 6	7.1	0.2	5.8	0.17	6.0*	0.22	5.1*	0.16	5.9*	0.26	4.7*	0.26
	Week 12	7.0	0.3	5.8	0.25	5.8*	0.20	4.9*	0.15	5.6*	0.28	4.6*	0.27

*p <0.05 compared to respective No-Intervention control group

Serum UA concentrations were similar at baseline (Table 1), in all the groups (*p* > 0.05). In a male-to-male and female-to-female comparison, the data in Table 2 show that in PA group, at week-6, males had -15 % and females had -12 % lower UA than those in NI (*p* < 0.05). At week-12, male and female participants of PA had -17 and -16 % lower concentrations, respectively, those in NI (*p* < 0.05). Table 2 also shows that, at week-6, serum UA in male and female participants of AA were -17 and -19 % less than in NI (*p* < 0.05). At week-12, UA was further reduced up to -20 and -21 % in males and females, respectively, in AA.

**Fig. 1:**
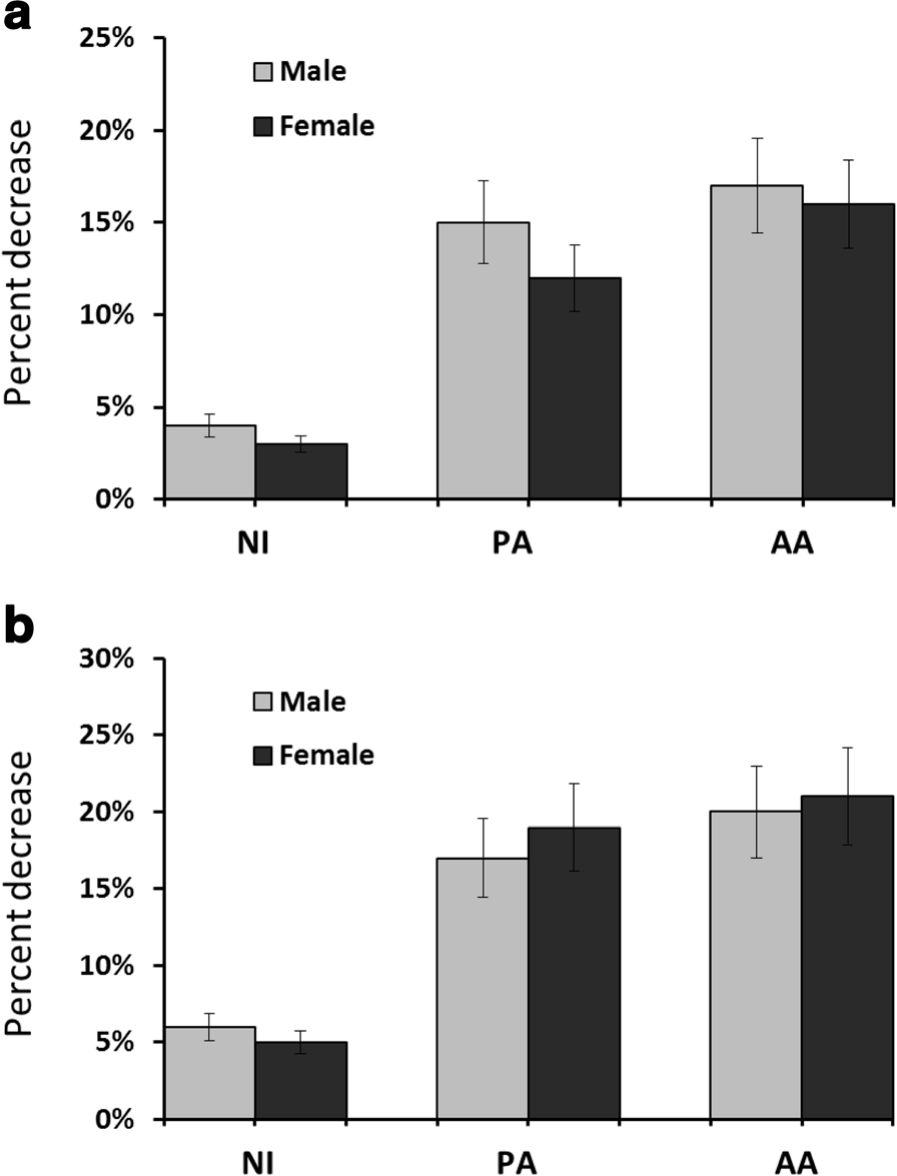
Percentage change in serum uric acid (UA) from baseline, at week-6 (**a**) and week-12 (**b**), in coronary artery disease patients randomized in no-intervention (NI), Pakistani almond (PA) or American almond (AA) groups. Values are mean ± SEM; *n* = 34 in NI group, *n* = 38 in PA group and *n* = 41 in AA group

Figure 1a shows comparison with respect to baseline. At week-6, drop in serum UA concentration in male (1 %) and female (2 %) participants of NI was insignificant (*p* > 0.05). In the almonds-intervention groups, there was a significant (*p* < 0.05) drop in UA; around -13 % in males of both PA and AA, and -11 and -16 % in females of PA and AA, respectively.

Figure 1b compares UA concentrations of baseline and intervention. At week-12, there was negligible (-3 and -2 %) drop in males and females of NI, while the drop in PA and AA was significant (-16 and -14 % in males and females of PA and -18 % in both males and females of AA; *p* < 0.05).

## Discussion

To the best of our knowledge, this is the first almond-intervention study on CAD patients reporting UA reduction. Previously, in rat model, dietary almonds prevented high-fat diet-induced hyperuricemia, followed by reduced nitric oxide (NO) production via endothelial NO synthase (eNOS) inhibition resulting in improved vascular function of isolated aorta [13]. So, from results in CAD patients, we can also infer probable vascular protection.

Serum UA may be considered as a biomarker for vascular function [15]. Anticipated pathways of UA-induced vascular dysfunction [16] include, but are not limited to: pro-oxidative affect, whereby UA decomposes and generates free radicals; pro-inflammatory affect via association with biomarkers like interleukins (IL-1, IL-6, IL-10, IL-18), tumor necrosis factor (TNF-α) and C-reactive proteins (CRP); endogenous stimulation of innate immunity; changing expression of endothelin-1; promoting angiotensin-II production; inducing smooth muscle cell proliferation; and direct reaction with, and depletion of NO.

Almond supplementation is shown to influence some of these parameters. Although, Jenkins et al. report no effect of almonds on CRP, BP or pulmonary NO [17]; Rajaram et al. [18] observed significant reduction in CRP. Two studies in diabetic patients [19, 20], report a drop in CRP accompanied by anti-inflammation via IL-6 reduction; while Liu et al. [20], also reports decrease in TNF-α by dietary almonds. Bhardwaj et al. [21], also demonstrated drop in CRP, accompanied by improved flow-mediated dilatation, indicating improved endothelial function. But more recently, Chen et al. [22] reported no effect of almond supplementation on vascular function, CRP, TNF-α and even the lipid profile of CAD patients in a randomized cross-over clinical trial. This may be due to the drug therapy which could mimic the effect of intervention. Decrease in vascular cell adhesion molecules was observe, which may indicate improvement in vascular function. Trials longer than four to six weeks may be able to offer detectible improvements in vascular function of CAD patients.

Almonds are rich in L-arginine, which is a precursor of NO. Supplementation of L-synthetic arginine reverses hyperuricemia-induced hypertension in rats [23]. However, BP of participants in our trial remained fairly constant during twelve weeks. Reason being, almost all CAD patients were on anti-hypertensive medications, and baseline BP was within normal ranges.

The precise underling mechanisms of almonds action on serum uric acid remains to be explored. Certain limitations of our trial include the following. The inclusion criteria included only those CAD patients, who had optimal LDL-C and sub-optimal HDL-C. This may not truly represent the CAD population in general. Sample size was calculated for observable improvements in serum HDL.

## Conclusion

Almond supplementation can provide holistic benefits to CAD patients. Previous clinical studies have almonds’ potential in ameliorating dyslipidemia, the current investigation adds to its vasculo-protective effects, by presenting prominent reduction in serum uric acid.

## Abbreviations

AA, American almonds; BP, blood pressure; CAD, coronary artery disease; CVD, cardiovascular diseases; NI, no intervention; NO, nitric oxide; PA, Pakistani almonds; UA, uric acid
